# Respiratory and Urinary Tract Infections, Arthritis, and Asthma Associated with HTLV-I and HTLV-II Infection

**DOI:** 10.3201/eid1001.020714

**Published:** 2004-01

**Authors:** Edward L. Murphy, Baoguang Wang, Ronald A. Sacher, Joy Fridey, James W. Smith, Catharie C. Nass, Bruce Newman, Helen E. Ownby, George Garratty, Sheila T. Hutching, George B. Schreiber

**Affiliations:** * University of California San Francisco, California, USA; † Westat, Rockville, Maryland, USA; ‡ Hoxworth Blood Center, Cincinnati, Ohio, USA; § Blood Bank of San Bernardino and Riverside Counties, San Bernardino, California, USA; ¶ Oklahoma Blood Institute, Oklahoma City, Oklahoma, USA; # American Red Cross Blood Services Chesapeake and Potomac, Baltimore, Maryland, USA; ** American Red Cross Blood Services Southeastern Michigan, Detroit, Michigan, USA; †† American Red Cross Blood Services Southern California, Los Angeles, California, USA

## Abstract

Human T-lymphotropic virus types I and II (HTLV-I and -II) cause myelopathy; HTLV-I, but not HTLV-II, causes adult T-cell leukemia. Whether HTLV-II is associated with other diseases is unknown. Using survival analysis, we studied medical history data from a prospective cohort of HTLV-I– and HTLV-II–infected and –uninfected blood donors, all HIV seronegative. A total of 152 HTLV-I, 387 HTLV-II, and 799 uninfected donors were enrolled and followed for a median of 4.4, 4.3, and 4.4 years, respectively. HTLV-II participants had significantly increased incidences of acute bronchitis (incidence ratio [IR] = 1.68), bladder or kidney infection (IR = 1.55), arthritis (IR = 2.66), and asthma (IR = 3.28), and a borderline increase in pneumonia (IR = 1.82, 95% confidence interval [CI] 0.98 to 3.38). HTLV-I participants had significantly increased incidences of bladder or kidney infection (IR = 1.82), and arthritis (IR = 2.84). We conclude that HTLV-II infection may inhibit immunologic responses to respiratory infections and that both HTLV-I and -II may induce inflammatory or autoimmune reactions.

Human T-lymphotropic virus types I and II (HTLV-I and -II) are presumed to have derived from primate T-lymphotropic viruses with which they share significant nucleotide sequence homology (*1*). They are transmitted by sexual intercourse; by parenteral modes such as unscreened blood or shared injection equipment; and from mother to child, predominantly by breast feeding (*2*–*4*). HTLV-I has been causally associated with adult T-cell leukemia and HTLV-associated myelopathy. HTLV-II has also been associated with HTLV-associated myelopathy, but not with leukemia (*5*).

Other possible health outcomes of chronic HTLV-I and -II infection have not yet been adequately investigated. Patients with adult T-cell leukemia may develop opportunistic infections such as Pneumocystis carinii pneumonia (*6*) or Strongyloides superinfection (*7*), but clinical immunodeficiency does not appear to develop in most persons with chronic HTLV-I or -II infection. On the contrary, syndromes suggestive of increased immunologic response such as uveitis (*8*), pneumonitis (*9*,*10*), and rarely, cases of lymphocytic arthritis (*11*,*12*) have been reported, although only uveitis has been epidemiologically associated with HTLV-I (*8*). Investigators in Japan have linked HTLV-I to a higher occurrence of various medical conditions (*13*) and virus-associated malignancies (*14*). Other investigators have reported an association between HTLV-II and pneumonia among injection drug users (*15*).

Case series and cross-sectional studies of HTLV-I and -II disease outcomes are vulnerable to potential bias and confounding. We have prospectively followed a large cohort of former blood donors with well-documented HTLV-I and -II infection at enrollment, and a similar group of uninfected donors, all of whom are HIV seronegative. We report on the occurrence of various disease outcomes in this cohort after a median follow-up of 4.3 years.

## Methods

### Study Design and Participants

This study is a prospective, multicenter cohort of persons with HTLV-I and -II infections, which were detected at the time of attempted blood donation at five U.S. blood centers and comparable HTLV–seronegative donors. Details of the cohort enrollment and follow-up procedures have been published previously (*16*,*17*). The study protocol was approved by the USCG committe on Human Research and by IRBs at other participating institituions

We determined HTLV serostatus by obtaining enzyme immunoassay test results followed by confirmatory Western blot. A central laboratory performed HTLV-I versus -II typing with a type-specific serologic assay, polymerase chain reaction (PCR), or both, as previously described (*18*). Unequivocal results from the type-specific serologic assay correlated well with those from the polymerase chain reaction assay. All participants were seronegative for HIV when baseline test were performed. For most participants, hepatitis C virus antibody status was not available at the time of enrollment.

### Disease Endpoints

Each visit with a study nurse consisted of an interviewer-administered health history questionnaire and phlebotomy of blood for complete blood count and other studies. Selected diagnoses (cancer, neurologic and autoimmune conditions) reported on the questionnaire triggered requests for confirmatory medical records. We included nine conditions or diseases (pneumonia, acute bronchitis, bladder or kidney infection, arthritis, hypertension, asthma, cancer, diabetes, and thyroid disease) and eight symptoms (trouble walking, climbing, or rising from chair; incontinence; pre- or post-void urgency; lymphadenopathy; night sweats; weight loss; foot paresthesias; and impotence [males]) in the data analysis.

### Statistical Analysis

We used the Kaplan-Meier product limit method to calculate the unadjusted probability of disease-free survival during the study period for each disease outcome by HTLV status. Survival time was defined as the number of days from the baseline visit until the date that an adverse health outcome was first diagnosed or the end of observation. We performed the log-rank test to assess the differences in disease‑free survival time (days) between HTLV-seronegative participants and HTLV‑I– or HTLV-II– infected participants, respectively.

To adjust for possible confounding factors, we performed multivariable analysis with HTLV status as an independent variable, survival time as a dependent variable, and possible confounding factors as covariates. In constructing the survival analysis models, we considered a number of covariates, which are described as follows: demographic variables (forced into all models), education, smoking history (pack-years, forced into the models for bronchitis and pneumonia), alcohol consumption, blood center, community versus autologous donation type, injection drug use (except in models for arthritis, hypertension, cancer, neurologic and urologic symptoms), parity (in models for urinary symptoms, bladder and kidney infections, and in females only), and number of sex partners (in model for bladder and kidney infections only). Using a backward selection process, all these covariates were added to the initial model, but only covariates with significant independent associations themselves (p < 0.05), or which substantively modified HTLV incidence ratio, were retained in the final model. We did not include interaction terms because similar analyses of data from prior cohort visits had indicated the absence of significant interaction.

To examine the differences in the cumulative number of episodes of pneumonia, bronchitis, and bladder or kidney infection by HTLV status, we used the negative binomial model, a generalization of the Poisson model, to compute incidence rate ratios (RR) with 95% confidence interval (CI) for each of these outcomes. This model took into account that the recurrence of the disease in a participant may be associated with both the overall disease incidence and its previous occurrence in that participant. We also adjusted for possible confounding factors using the same modeling strategy as for the survival analyses.

For the analysis of symptoms, we calculated unadjusted and adjusted odds ratio (OR) with 95% CI for any occurrence of each symptom by HTLV status by using logistic regression models. To adjust for possible confounding, we added other possible confounding factors as covariates to the models by using the same approach as in the survival analyses. All analyses were performed using Statistical Analysis System (SAS) software (*19*).

## Results

### Study Population and Follow-up

During the initial visits in 1990 through 1992, we enrolled 154 HTLV-I, 387 HTLV-II, and 799 HTLV seronegative persons. Two HTLV-I participants were excluded from this analysis because they did not complete the screening physical examination at the initial visit. The baseline characteristics of the study population are given in Table 1. The HTLV groups and seronegative participants were comparable with respect to age, sex, race or ethnicity, blood center visited, and type of blood donation (autologous versus allogeneic), except for slightly higher proportions of blacks among the HTLV-I group and Hispanics among the HTLV-II group. The HTLV-II group had the lowest socioeconomic status, as indicated by educational attainment and income, and the HTLV-I group had intermediate status. Pack-years of cigarette smoking and amount of alcohol intake were higher in the HTLV-II group. Consistent with the recognized epidemiologic risk factors for HTLV-I and -II infection (*3*,*4*), the HTLV-I and -II groups had more lifetime sexual partners than seronegative participants, and almost 24% of the HTLV-II participants had a lifetime history of injection drug use, although only 4.4% of the HTLV-II group admitted to current injection drug use.

**Table 1:** Characteristics of the multicenter, prospective human T-lymphotropic virus (HTLV) cohort study population^a^

Characteristics	**HTLV-I** **(n = 152)**	**HTLV-II** **(n = 387)**	**HTLV negative** **(n = 799)**
Age (y)	No. (%)	No. (%)	No. (%)
18–29	5 (3)	11 (3)	34 (4)
30–39	28 (18)	104 (27)	171 (21)
40–49	55 (36)	168 (43)	288 (36)
50–59	32 (21)	73 (19)	175 (22)
>60	32 (21)	31 (8)	131 (16)
Sex			
Male	43 (28)	102 (26)	257 (32)
Female	109 (72)	285 (74)	542 (68)
Race/ethnicity			
Asian	20 (13)	8 (2)	60 (8)
Black	61 (40)	125 (32)	248 (31)
Hispanic	9 (6)	104 (27)	150 (19)
Other	1 (1)	7 (2)	30 (4)
White	59 (39)	140 (36)	309 (39)
Unknown	2 (1)	3 (1)	2 (0)
Education			
High school or less	45 (30)	135 (35)	129 (16)
Some college	66 (43)	195 (51)	363 (46)
College	30 (20)	45 (12)	181 (23)
College (>4 years)	11 (7)	11 (3)	123 (15)
Income			
<$30,000	46 (30)	144 (38)	167 (21)
$30,000–49,999	51 (34)	120 (32)	221 (28)
>$50,000	55 (36)	113 (30)	401 (51)
Center			
1	32 (21)	51 (13)	122 (15)
2	29 (19)	39 (10)	102 (13)
3	44 (29)	206 (53)	345 (43)
4	31 (20)	68 (18)	156 (20)
5	16 (11)	23 (6)	74 (9)
Blood donor type			
Autologous	28 (18)	39 (10)	111 (14)
Allogeneic	124 (82)	348 (90)	688 (86)
Smoking history (pack/y)			
Nonsmoker	74 (52)	125 (36)	413 (54)
0–13	24 (17)	117 (33)	184 (24)
>13	43 (31)	109 (31)	174 (23)
Alcohol intake (average drinks per wk)			
Nondrinker	19 (13)	20 (6)	70 (9)
0–1	58 (41)	134 (38)	352 (46)
>1	64 (45)	200 (57)	339 (45)
Lifetime sex partners			
<6	56 (38)	87 (23)	381 (49)
>6	92 (62)	292 (77)	403 (51)
Injection drug use			
Ever	148 (98)	294 (76)	787 (99)
Ex-injection drug user	2 (1)	75 (19)	9 (1)
Current injection drug user	1 (1)	17 (4)	1 (0)

^a^Missing data (up to 6%, depending upon the variable) were excluded from the calculation of percentages.

We present data through the third study visit in 1995 through 1996. Median follow-up time was 4.3 years for all 1,338 participants, including those with no follow-up. Median follow-up did not differ by HTLV status and was 4.4 years for the HTLV-I group, 4.3 years for the HTLV-II group, and 4.4 years for the HTLV-seronegative group.

### HTLV-I Findings

Compared to seronegative persons, HTLV-I–infected persons were more likely to have a new diagnosis of bladder or kidney infection (p = 0.009) and arthritis (p = 0.0002) (Figure). Among the HTLV-I–infected persons, two had asthma; an insufficient number to test the difference relative to seronegative persons. The HTLV-I participants showed no statistically significant differences in the incidence of pneumonia, acute bronchitis, and hypertension, cancer, diabetes, and thyroid disease (data not shown).

**Figure:**
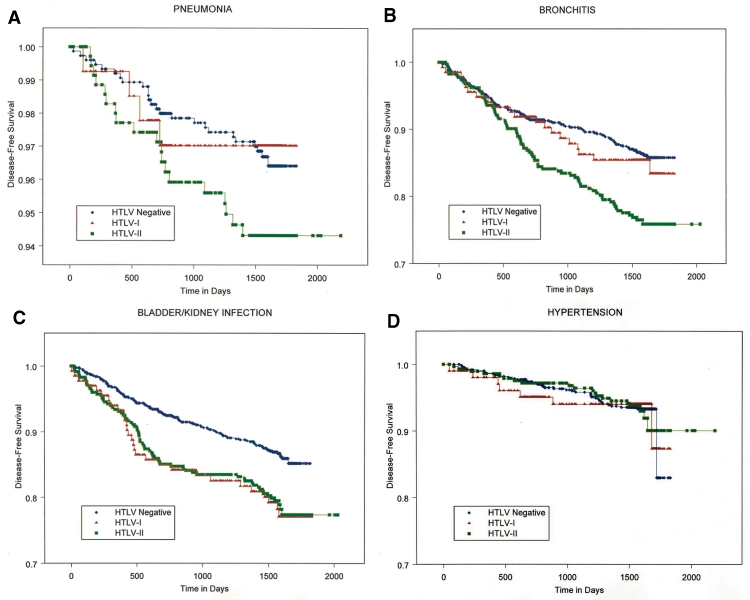
Kaplan-Meier survival curves showing disease-free survival for one noninfectious and three infectious diseases, by human T-lymphotropic virus (HTLV) status, through visits 2 and 3 of prospective observation. HTLV-I–infected (triangles) and HTLV-II–infected (squares) participants are compared to HLTV-seronegative participants (diamonds), respectively. Panels are as follows: A) pneumonia; B) acute bronchitis; C) bladder or kidney infection; and D) hypertension. The vertical axis scale has been compressed because of the lower overall incidence of pneumonia.

The number of incident cases diagnosed (limited to one case per person) and the unadjusted and adjusted incidence ratios (IR) for several diagnoses are given in Table 2. Compared with results for seronegative persons, and after multivariable adjustment for relevant confounding variables, HTLV-I infection was associated with bladder or kidney infection (IR 1.82, 95% CI 1.19 to 2.77) and arthritis (IR 2.84, 95% CI 1.51 to 5.33). The risks of developing pneumonia, acute bronchitis, hypertension, and cancer were not significantly increased. Too few cases of asthma (n = 2), thyroid disease (n = 1), and diabetes mellitus (n = 5) existed among the HTLV-I participants to perform survival analysis.

**Table 2:** Incidence of medically diagnosed conditions and selected unadjusted and adjusted incidence ratios among human T-lymphotropic virus (HTLV)-I– and HTLV-II– infected participants and HTLV-seronegative participants, visits 2 and 3^a^

Diagnosis	HTLV seronegative (N = 799)	HTLV-I (N = 152)			HTLV-II (N = 387)		
	Cases (%^b^)	Cases (%^b^)	IR^c^	Adj. IR (95% CI)^d^	Cases (%^b^)	IR^c^	Adj. IR (95% CI)^d^
Pneumonia	25 (3)	5 (4)	0.89	0.79 (0.27 to 2.29)	19 (5)	1.70	1.82 (0.98 to 3.38)
Acute bronchitis	103 (14)	21 (15)	1.12	1.10 (0.68 to 1.79)	81 (23)	1.83	1.68 (1.24 to 2.29)
Bladder or kidney infection	105 (14)	31 (23)	1.74	1.82 (1.19 to 2.77)	73 (21)	1.68	1.55 (1.14 to 2.11)
Arthritis	32 (5)	16 (16)	3.19	2.84 (1.51 to 5.33)	32 (12)	2.51	2.66 (1.58 to 4.45)
Hypertension	40 (7)	7 (7)	1.06	0.99 (0.44 to 2.22)	20 (7)	1.08	1.09 (0.63 to 1.89)
Asthma	15 (2)	2 (2)	—	—	20 (6)	3.38	3.28 (1.57 to 6.84)
Cancer	21 (3)	3 (2)	0.81	0.72 (0.21 to 2.43)	8 (2)	0.87	1.10 (0.44 to 2.32)

^a^Only the first diagnosis of each condition is considered for each participant. IR, incidence ratio derived from survival analysis; CI, confidence interval. ^b^Denominator for percentage calculation varied according to the number of participants included in each disease-specific analysis. ^c^Unadjusted incidence ratio derived from survival analysis. ^d^After a backward selection process, including all potential confounding variables, the final survival analysis model contained these variables age, gender, race/ethnicity, and (for pneumonia, bronchitis, and asthma) smoking history (see Methods for details of the statistical analysis).

To further investigate the occurrence of infectious diseases, we also analyzed the incidence density of three infectious diseases by HTLV status, whether or not each diagnosis was a first or recurrent case (Table 3). In the incidence density analysis (Table 3), an average of 1.75 cases of bladder or kidney infection occurred per HTLV-I participant over the 4.4-year median follow-up time, compared with 0.63 per seronegative participant over the same period. The unadjusted and adjusted RRs for the HTLV-I group were significantly greater than unity for bladder or kidney infection. HTLV-I participants had increased prevalence rates of neurologic symptoms, self-reported lymphadenopathy, and night sweats, but they reported weight loss no more frequently than did HTLV-seronegative persons (Table 4).

**Table 3:** Incidence density (ID)^a^ and standard deviation (SD) of medically diagnosed infectious diseases, and selected crude and adjusted rate ratios (RR), among human T-lymphotropic virus (HTLV)-I– and HTLV-II–infected participants and HTLV-seronegative participants, visits 2 and 3

Diagnosis	HTLV seronegative (N = 799)	HTLV-I (N = 152)			HTLV-II (N = 387)		
	ID (SD)	ID (SD)	RR^b^	Adj. RR (95% CI)^c^	ID (SD)	RR^b^	Adj. RR (95% CI)^c^
Pneumonia	0.08 (0.37)	0.11 (0.47)	1.49	1.33 (0.66 to 2.66)	0.21 (0.96)	2.82	2.65 (1.67 to 4.21)
Acute Bronchitis	0.59 (1.78)	0.82 (2.25)	1.38	1.33 (0.84 to 2.12)	1.10 (2.75)	1.83	1.53 (1.10 to 2.14)
Bladder or Kidney infection	0.63 (2.04)	1.75 (4.61)	2.73	2.32 (1.50 to 3.59)	1.25 (3.48)	1.94	1.94 (1.40 to 2.68)

^a^Defined as the mean of the total number of infectious disease diagnoses divided by the number of participants (with or without the infection) in each group at baseline. The period of observation was 4.4 years (HTLV-I), 4.3 years (HTLV-II), and 4.4 years (HTLV seronegative), and each participant may have multiple diagnoses of each condition.^b^Unadjusted. ^c^Adjusted. Adjusted models included age, gender, race/ethnicity and duration of follow-up for all infections, and injection drug use (for pneumonia), smoking (for acute bronchitis), and community versus autologous blood donation (for bladder/kidney infection).

**Table 4:** Prevalence of medically diagnosed symptoms, and unadjusted and adjusted odds ratios (OR) derived from logistic regression models, in human T-lymphotropic virus (HTLV)-I– and HTLV-II–infected participants and HTLV-seronegative participants, visits 2 and 3^a^

Symptoms	HTLV seronegative (N = 799)	HTLV-I (N = 152)			HTLV-II (N = 387)		
	Cases (%)^b^	Cases (%)^b^	OR^c^	Adj. OR (95% CI)^d^	Cases (%)^b^	OR^c^	Adj. OR (95% CI)^d^
Trouble walking, climbing or rising from chair	147 (21)	52 (42)	2.71	2.67 (1.74 to 4.09)	133 (42)	2.78	3.44 (2.52 to 4.71)
Incontinence, pre- or post-void urgency	175 (25)	50 (40)	2.03	2.02 (1.33 to 33.07)	134 (43)	2.25	2.59 (1.92 to 3.49)
Lymphadenopathy	29 (4)	11 (9)	2.26	2.39 (1.14 to 5.03)	40 (13)	3.40	3.08 (1.85 to 5.13)
Night sweats	20 (3)	15 (12)	4.68	4.73 (2.31 to 9.69)	47 (15)	6.02	4.97 (2.77 to 8.94
Weight loss	40 (6)	9 (7)	1.29	1.10 (0.51 to 2.37)	40 (13)	2.43	2.10 (1.22 to 3.60)
Foot paresthesias	57 (8)	22 (18)	2.44	2.46 (1.41 to 4.28	66 (21)	3.02	3.27 (2.19 to 4.88)
Impotence (males only)	33 (5)	10 (8)	2.24	2.05 (0.77 to 5.49)	13 (4)	1.10	1.27 (0.56 to 2.91)

^a^Only the first diagnosis of each symptom is considered for each participant. ^b^Denominator for percentage calculation varied according to the number of participants included in each disease-specific analysis. ^c^Unadjusted. ^d^Adjusted. Models were adjusted for age, race and ethnicity, and duration of follow-up (all symptoms), and gender (all except impotence). In addition, specific models included community versus autologous donation (for trouble walking and incontinence, weight loss and impotence.) and injection drug use [night sweats and weight loss]).

### HTLV-II Findings

Disease-free survival curves for selected medical diagnoses, by HTLV status, are represented in the Figure. Compared to seronegative persons, HTLV-II–infected participants were more likely to experience acute bronchitis (p < 0.0001), bladder or kidney infection (p = 0.0008), arthritis (p = 0.0003), and asthma (p = 0.0007); the likelihood of acquiring pneumonia was increased but not significantly (p = 0.08). Differences between the HTLV-II and HTLV-seronegative participants in the incidence of cancer, hypertension, diabetes, or thyroid disease were not statistically significant.

The number of incident cases diagnosed (limited to one case per participant), and the unadjusted and adjusted IRs, for several diagnoses are given in Table 2. Compared with results for seronegative participants, and after adjusting for confounding variables, HTLV-II was associated with acute bronchitis (OR 1.68, 95% CI 1.24 to 2.29), bladder or kidney infection (OR 1.55, 95% CI 1.14 to 2.11), arthritis (OR 2.66, 95% CI 1.58 to 4.45), and asthma (OR 3.28, 95% CI 1.57 to 6.84). The association between HTLV-II infection and pneumonia was of borderline statistical significance (IR 1.82 , 95% CI 0.98 to 3.38). IRs for hypertension and cancer were not increased for HTLV-II participants. Too few cases of thyroid disease (n = 7) and diabetes mellitus (n = 7) were found among the HTLV-II participants to perform survival analysis.

In the incidence density analysis of recurrent infections, an average of 0.21 cases of pneumonia, 1.10 cases of acute bronchitis, and 1.25 cases of bladder or kidney infection occurred among HTLV-II participants during their 4.3-year median follow-up time (Table 3). The corresponding incidence densities for the seronegative group were 0.08, 0.59, and 0.63, respectively, over their 4.4 year median follow-up time. Both unadjusted and adjusted RRs, calculated by using negative binomial modeling, were significantly greater than unity for all three diseases (Table 3). Compared with seronegative participants, HTLV-II participants had increased prevalence rates of several neurologic, lymphatic, and constitutional symptoms, but the prevalence of impotence (males) was not significantly increased (Table 4).

## Discussion

In summary, HTLV-II–infected participants had a higher incidence of acute bronchitis, bladder or kidney infection, arthritis and asthma, and a higher incidence of pneumonia than did HTLV-seronegative participants followed concurrently. The finding of a higher rate of these infectious diseases among HTLV-II participants was supported both by survival analysis, which considered only the first diagnosis, and by negative binomial modeling, which considered both first and recurrent infections. HTLV-I participants had a higher incidence of bladder or kidney infection and arthritis. Cancer incidence was not higher in either the HTLV-I or HTLV-II participants compared to its incidence in seronegative participants, although the number of cases was small.

Our finding of a higher incidence of respiratory tract infections among HTLV-II–infected persons is consistent with most of the small number of other published clinical studies on this topic. Robert-Guroff et al. found higher rates of HTLV-II infection among injection drug users with abscess than in those without abscess (*20*). In another cross-sectional analysis, Modahl et al. found that injection drug users with HTLV-II infection were more likely to have been diagnosed with pneumonia, abscess, or lymphadenopathy (*15*), although a subsequent case-control study did not find that either HTLV-II or HIV are risk factors for skin and soft tissue abscess among injection drug users (*21*). LaGrenade et al. have documented an association between HTLV-I infection and Staphylococcus- and Streptococcus-related infective dermatitis among Jamaican children (*22*). An independent nested case-control study of pneumonia, abscess, and endocarditis among Baltimore injection drug users found no association between these infections and HTLV-II seropositivity (*23*). A number of opportunistic co-infections have been reported in conjunction with HTLV-I infection, including Strongyloides hyperinfection (*7*), P. carinii pneumonia (in patients with HTLV-I–related adult T-cell leukemia) (*6*), and leprosy (*24*).

The biologic basis for a putative increased susceptibility to certain infections in humans with chronic HTLV-II infection is not well described. In contrast to the predominant CD4+ lymphotropism of HTLV-I, HTLV-II provirus in vivo is integrated at highest levels into CD8+ lymphocytes but may also be demonstrated in CD4+, both CD45RO+ and CD45RO-, and even non-T (CD14, CD16, and CD19) lymphocytes (*25*). Delayed hypersensitivity response to mumps virus and Candida antigens is normal among HTLV-II participants, suggesting intact cell-mediated or T-helper 1-type immunity (*26*). Although subtle differences may exist, the overall distribution of lymphocyte subsets is not perturbed in persons with HTLV-II (*27*,*28*). However, total immunoglobulin G levels are higher in HTLV-II–infected persons (*29*), in vitro cell proliferation in response to pokeweed mitogen is suppressed in HTLV-II infection (*28*), and HTLV-II may induce expression of interferon-g, granulocyte macrophage–colony-stimulating factor, and other cytokines (*30*,*31*). Although HTLV-II provirus has also been demonstrated in macrophages (*32*), whether such infection influences macrophage regulation or function to a clinically notable degree is not known. Finally, lymphocytic pneumonitis has been reported in association with HTLV-I infection (*9*,*10*), and clinically diagnosed cases of pneumonia and acute bronchitis in HTLV-II– infected persons could conceivably represent autoimmune rather than infectious disease.

Our finding of an association of both HTLV-II and HTLV-I with bladder or kidney infection is consistent with a previous report of unspecified renal disease in a prospective cohort study of HTLV-I–infected persons in Japan (*33*). However, such associations must be interpreted cautiously in light of the known association of both retroviruses with HTLV-associated myelopathy (*5*,*34*,*35*). Since urinary frequency and urgency are among the first symptoms of bladder hyperreactivity due to the underlying myelopathy, HTLV-I– or -II–infected persons might seek medical care for these symptoms. Urinary tract infection or kidney disease secondary to unrecognized neurogenic bladder dysfunction might be diagnosed, or they may be treated presumptively for urinary tract infection on the basis of their bladder symptoms. In either case, an increased incidence of diagnosed bladder or kidney infection may not necessarily indicate that HTLV-I or -II infection is the cause of these urinary infections. Finally, although we controlled for the number of sexual partners, residual confounding by sexual activity could have influenced our bladder or kidney infection finding (*36*).

The increased incidence of arthritis observed for both persons infected with HTLV-I and HTLV-II supports reports of possible autoimmune syndromes with HTLV infection. HTLV-I has been epidemiologically associated with uveitis (*8*). Several previous reports of HTLV-I in case series of arthritis have been limited by the lack of appropriate controls (*11*,*12*). Nonetheless, high numbers of HTLV-I–infected lymphocytes have been demonstrated in synovial fluid from some of these case-patients. Although we obtained medical records to verify arthritis and other diagnoses, the records did not give sufficient information to classify the type of arthritis and diagnostic evaluations were limited in most cases. Although an association between HTLV-I infection and asthma has been reported among Japanese men (*13*), we are unaware of previous reports of an increased incidence of asthma in association with HTLV-II infection. Also, a few cases of lymphocytic pneumonitis have been reported in patients with HTLV-I infection, particularly those with myelopathy (*9*,*10*). We plan a more intensive diagnostic evaluation of the HTLV-II participants in this study with recurrent pneumonia or asthma to explore the possible contribution of undiagnosed lymphocytic pneumonitis to the observed clinical signs and symptoms.

We have previously reported a single case of adult T-cell leukemia which was diagnosed between the first and second visits of the patient in this cohort study (*17*); no additional cases have been diagnosed to date. That neither HTLV-I nor -II participants had an increased incidence of nonhematologic cancer in our current analysis is potentially reassuring to persons infected with these retroviruses. However, an increased incidence of some cancers, especially those thought to be induced by viruses, has been reported in a Japanese HTLV-I cohort (*37*). We might not have detected a small increase in IR because of the relatively small number of cases detected during our 4.3-year follow-up period. Alternatively, we might not have had enough HTLV-I or -II participants who were co-infected with other oncogenic viruses such as hepatitis C virus to detect a synergistic effect between HTLV-I or -II and these viruses (*38*).

The two- to three-fold higher prevalence of self-reported neurologic symptoms, including trouble walking, climbing stairs, or rising from a chair, and bladder symptoms may represent early spinal cord injury due to HTLV-I or -II. As follow-up of the cohort continues, we shall be able to determine whether those reporting such symptoms in earlier visits have a higher incidence of overt myelopathy than asymptomatic HTLV-infected participants. On the other hand, the frequency of self-reported lymphadenopathy and night sweats is unlikely to be caused by preclinical hematologic malignancy, given the rarity of that disorder in HTLV-I–infected persons and its lack of association with HTLV-II. These symptoms might be related to known effects of HTLV-I and -II on lymphocytic proliferation and cytokine expression, or they might simply reflect reporting bias.

Strengths of the current study include its controlled, prospective, cohort design, stringent confirmation of HTLV-I– and -II–infection status at baseline, and systematic ascertainment of disease outcomes. One potential weakness is that differences in socioeconomic status and risk behaviors could have confounded disease associations between HTLV–infected and uninfected previous blood donors, even though we selected the uninfected participants in strata defined by the age, sex, race or ethnicity, center, and blood donation type of the HTLV groups. We controlled for the socioeconomic and behavioral factors using multivariate analyses, but residual confounding could affect the magnitude of the associations we observed. Second, recall bias may exist in that participants with HTLV infection might differentially report more diagnoses because of heightened concern about their own health. Our questionnaire requested only medically confirmed diagnoses, and the absence of associations with noninfectious disease, such as hypertension, diabetes, and thyroid disease, suggests that generalized overreporting of illness was not a problem. Additionally, infectious disease associations with HTLV have not been widely reported, so we do not think that recall bias specific to these diagnoses was a serious concern. Finally, follow-up time to date is modest for a chronic infection such as HTLV, and our findings may change with longer observation.

In conclusion, HTLV-II infection is associated with an increased incidence of respiratory and urinary tract infections and asthma, and both HTLV-I and -II are associated with increased incidence rates of arthritis, compared with results for seronegative persons. These findings suggest that chronic infection with HTLV-II may inhibit host immunologic responses to infection, or more specifically, to respiratory infections. The arthritis and asthma results, and possibly the respiratory tract diagnoses, suggest that other inflammatory or autoimmune reactions may be induced by HTLV-I or HTLV-II infection. Additional in vitro and in vivo research on the immunologic consequences of HTLV infection is needed.
